# Serum 25-hydroxyvitamin D, mortality, and incident cardiovascular disease, respiratory disease, cancers, and fractures: a 13-y prospective population study^1^,^2^,^3^,^4^

**DOI:** 10.3945/ajcn.114.086413

**Published:** 2014-09-17

**Authors:** Kay-Tee Khaw, Robert Luben, Nicholas Wareham

**Affiliations:** 1 From the Department of Public Health and Primary Care, Institute of Public Health, School of Clinical Medicine (K-TK and RL) and the Medical Research Council Epidemiology Unit (NW), University of Cambridge, Cambridge, United Kingdom.

## Abstract

**Background:** Vitamin D is associated with many health conditions, but optimal blood concentrations are still uncertain.

**Objectives:** We examined the prospective relation between serum 25-hydroxyvitamin D [25(OH)D] concentrations [which comprised 25(OH)D_3_ and 25(OH)D_2_] and subsequent mortality by the cause and incident diseases in a prospective population study.

**Design:** Serum vitamin D concentrations were measured in 14,641 men and women aged 42–82 y in 1997–2000 who were living in Norfolk, United Kingdom, and were followed up to 2012. Participants were categorized into 5 groups according to baseline serum concentrations of total 25(OH)D <30, 30 to <50, 50 to <70, 70 to <90, and ≥90 nmol/L.

**Results:** The mean serum total 25(OH)D was 56.6 nmol/L, which consisted predominantly of 25(OH)D_3_ (mean: 56.2 nmol/L; 99% of total). The age-, sex-, and month-adjusted HRs (95% CIs) for all-cause mortality (2776 deaths) for men and women by increasing vitamin D category were 1, 0.84 (0.74, 0.94), 0.72 (0.63, 0.81), 0.71 (0.62, 0.82), and 0.66 (0.55, 0.79) (*P*-trend < 0.0001). When analyzed as a continuous variable and with additional adjustment for body mass index, smoking, social class, education, physical activity, alcohol intake, plasma vitamin C, history of cardiovascular disease, diabetes, or cancer, HRs for a 20-nmol/L increase in 25(OH)D were 0.92 (0.88, 0.96) (*P* < 0.001) for total mortality, 0.96 (0.93, 0.99) (*P* = 0.014) (4469 events) for cardiovascular disease, 0.89 (0.85, 0.93) (*P* < 0.0001) (2132 events) for respiratory disease, 0.89 (0.81, 0.98) (*P* = 0.012) (563 events) for fractures, and 1.02 (0.99, 1.06) (*P* = 0.21) (3121 events) for incident total cancers.

**Conclusions:** Plasma 25(OH)D concentrations predict subsequent lower 13-y total mortality and incident cardiovascular disease, respiratory disease, and fractures but not total incident cancers. For mortality, lowest risks were in subjects with concentrations >90 nmol/L, and there was no evidence of increased mortality at high concentrations, suggesting that a moderate increase in population mean concentrations may have potential health benefit, but <1% of the population had concentrations >120 nmol/L.

## INTRODUCTION

Vitamin D deficiency is a well-established cause of impaired bone mineralization that leads to conditions such as rickets and osteomalacia. In addition, vitamin D status has been associated with other health outcomes including osteoporotic fractures, cardiovascular disease, cancers, diabetes, respiratory diseases, and all-cause mortality (1–17). However, substantial debate continues as to what an optimal vitamin D status might be above low blood concentrations associated with deficiency bone diseases (1), and randomized trials of vitamin D supplementation have not provided conclusive evidence (1, 2, 5). An understanding of the dose-response relation between serum vitamin D concentrations in a general population of men and women and a range of health outcomes may help our understanding of how vitamin D may relate to health and inform future trial designs or public health recommendations.

## SUBJECTS AND METHODS

Participants were recruited from age-sex registers of general practices for a prospective population study of 25,639 men and women aged 40–79 y, 99.5% of whom were white, who were residents in Norfolk, United Kingdom, and first surveyed in 1993–1997 as part of the 10-country collaboration the European Prospective Investigation into Cancer and Nutrition (18).

Between 1997 and 2000, ∼15,000 participants attended a second clinic visit (19). At this visit, participants completed a detailed health and lifestyle questionnaire. A personal medical history of heart attack, stroke, cancer, asthma, bronchitis, and fractures; smoking history; alcohol intake; supplement use; physical activity; social class; and educational status were ascertained by using standard questionnaires (19–21). Participants were asked about their medical histories with the question “Has a doctor ever told you that you have any of the following?” followed by a list of conditions that included heart attack, stroke, cancer, asthma, bronchitis, and fractures. Prevalent conditions were defined as yes answers to the relevant condition. Smoking history was derived from yes or no responses to the questions “Have you ever smoked as much as one cigarette a day for as long as a year?” and “Do you smoke cigarettes now?” Alcohol consumption was derived from the question “How many alcoholic drinks do you have each week?” with 4 separate categories of drinks. A unit of alcohol (∼8 g) was defined as a half-pint of beer, cider, or lager; a glass of wine; a single unit of spirits (whisky, gin, brandy, or vodka); or a glass of sherry, port, vermouth, or liqueurs. Total alcohol consumption was estimated as the total units of drinks consumed in a week. Supplement use was ascertained by using the question “Have you taken any vitamins, minerals, or other food supplements regularly during the past year (such as vitamin C, vitamin D, iron, calcium, fish oils, primrose oil, betacarotene, etc)?”

Habitual physical activity was assessed by using 2 questions about occupational and other physical activity, and individuals were allocated to a physical activity index validated against heart-rate monitoring in one of 4 categories as follows: inactive, moderately inactive, moderately active, and active (21).

Social class was classified according to the Registrar General's occupation-based classification scheme into 5 main categories with social class I representing professionals, social class II representing managerial and technical occupations, social class III representing subdivision into nonmanual and manual skilled workers, social class IV representing partly skilled workers, and social class V representing unskilled manual workers. We also recategorized social class into manual and nonmanual social classes. Social classes I–III nonmanual were classified as nonmanual, whereas social classes III manual, IV, and V were classified as manual (20). Educational status was based on the highest qualification attained and was categorized into 4 groups as follows: degree or equivalent, A-level or equivalent, O-level or equivalent, and less than O-level or no qualifications. O-level indicates educational attainment to the equivalent of completion of schooling to the age of 15 y, and A-level indicates educational attainment to the equivalent of the completion of schooling to the age of 17 y. Educational level was categorized into “at least O-level” (which includes O-level, A-level, and degree) and “no qualifications” (20).

Individuals with prevalent conditions were identified if they answered yes to the question “Have you been diagnosed by a doctor as having any of the following conditions? followed by a checklist of the individual conditions previously listed and a box for filling in dates and more details. Dietary intake was assessed by using a mailed food-frequency questionnaire and analyzed for nutrient intake including dietary calcium (22).

Trained nurses carried out a health examination. BMI (in kg/m^2^) was estimated as weight divided by the square of height. Plasma and serum samples were obtained from venipuncture blood samples. The plasma vitamin C concentration as a biomarker of plant food intake was estimated by using a fluorometric assay ≤1 wk of sampling (23, 24). Samples were stored frozen in liquid nitrogen tanks until 2012 when serum samples were retrieved for 25-hydroxyvitamin D [25(OH)D] assays. Assays were conducted by VITAS, which is a reference laboratory in Nordic countries for fat soluble vitamins (25–27). Assays for 25(OH)D were based on ultraperformance liquid chromatography interfaced by atmospheric pressure chemical ionization to mass spectrometry. This method measures the C3 epimer 3-epi-25(OH)D_3_ as well as 25(OH)D_3_ and 25(OH)D_2_. The limit of detection is 1–4 nmol/L. CVs for interassay analyses are 7.6% at 25(OH)D concentrations of 47.8 nmol/L and 6.9% at 83.0 nmol/L.

All participants were followed up for health events. We report results for follow-up to December 2012 (an average of 13 y). All participants were flagged for death certification at the Office of National Statistics, United Kingdom. Death certificates were coded by nosologists according to the International Classification of Diseases (ICD). An underlying cause of death was defined by using ICD codes as follows: for cardiovascular death (ICD9 400–438 or ICD10 I10–I79), cancer death (ICD9 140–208 or ICD10 C00–C97), or respiratory disease (ICD9 460–519 or ICD10 J00–J99). We also examined specifically the following 2 subgroups: chronic respiratory disease ICD9 490–496 or ICD10 J40–J47 and respiratory infections ICD9 466 and 480–487 or ICD10 J10–J22 and J85. Deaths that were not attributable to underlying cardiovascular, respiratory, or cancer causes were classified as deaths from other causes. All participants are also linked to the National Cancer Registry for incident cancers. Participants admitted to hospital were identified by their unique national health service number by using data linkage with ENCORE (East Norfolk Health Authority database), which identifies all hospital contacts throughout England and Wales for Norfolk residents. We used diagnostic codes to ascertain dates of first-incident hospital admissions for cardiovascular diseases, respiratory diseases, and fractures. We analyzed data according to deaths by underlying cause and total incident fatal and nonfatal diseases, which were defined as either death or hospital admission for the relevant condition, which is an approach that was previously validated for cardiovascular conditions by using medical records. The study was approved by the Norwich District Health Authority Ethics Committee, and all participants gave signed informed consent.

The current analysis included 14,641 men and women aged 42–82 y who attended the second health examination in 1997–2000 and had available blood samples for assays. Total 25(OH)D was calculated as the sum of 25(OH)D_3_ and 25(OH)D_2_. Where concentrations of 25(OH)D_2_ were below limits of detection (1 nmol/L), they were coded as zero. We categorized total 25(OH)D into 5 categories by using clinically relevant cutoffs as follows: <30, 30 to <50, 50 to <70, 70 to <90, and ≥90 nmol/L (multiply by 0.4 to get ng/mL).

We examined risk-factor distributions in men and women by 25(OH)D category. Concentrations of 25(OH)D showed marked seasonal variations, and thus, all results were adjusted for the month of assessment. The Cox proportional hazards model was used to determine RRs of all-cause and cause-specific mortality, incident cancers, and total fatal and nonfatal incident cardiovascular disease, respiratory diseases, and fractures by plasma 25(OH)D category after adjustment for age, sex, and month and then further adjustment for BMI, cigarette-smoking habit, alcohol intake, physical activity, plasma vitamin C, social class, education, and history of cardiovascular disease, cancer, or diabetes. We estimated HRs for all-cause mortality, incident total cancers, and incident chronic disease hospitalizations by using plasma vitamin D as a continuous variable per 20-nmol/L increase. We also examined RRs in subgroups stratified by sex, age group, BMI, manual and nonmanual social classes, smoking status, physical activity, and any supplement use and after the exclusion of individuals with any history of cardiovascular disease, diabetes, cancer, fractures, or respiratory diseases and those who died within ≤2 or ≤5 y of follow-up. We compared mean baseline 25(OH)D concentrations by the month of assessment in subjects who survived and those who died. To enable the categorization of individuals by taking into account the seasonal variation, we estimated coefficients according to the month of blood draw in relation to the estimate in November, which was the month that most closely approximated the overall individual annual mean.

We also compared survival curves in individuals with concentrations >120 nmol/L to examine mortality in subjects with highest vitamin D concentrations in this cohort although they accounted for only 1% of the total cohort. Only 21 individuals (0.1%) had 25(OH)D concentrations >150 nmol/L, and 2 individuals had 25(OH)D concentrations >80 nmol/L in this cohort.

## RESULTS

Of 14,641 individuals with 25(OH)D_3_ results, only 925 individuals had measurable 25(OH)D_2_ concentrations, and 8736 individuals had measurable 3-epi-25(OH)D_3_ concentrations. Population mean concentrations were 56.2 nmol/L (range: 3.4–201.9 nmol/L) for 25(OH)D_3_, 0.35 nmol/L (5.6 nmol/L in those with detectable concentrations) (range: 0–82.5 nmol/L) for 25(OH)D_2_, and 1.36 nmol/L (2.3 nmol/L in those with detectable concentrations) (range: 0–46.3 nmol/L) for 3-epi-25(OH)D_3_. The total 25(OH)D concentration, which was calculated as the sum of 25(OH)D_3_ and 25(OH)D_2_, had a mean of 56.6 nmol/L (range: 4.6–201.9 nmol/L) with 25(OH)D_3_ contributing 99% of the plasma total, and 25(OH)D_2_ contributing 1% of the plasma total.

Characteristics of participants by 25(OH)D category measured in 1997–2000 are shown in **Table 1**. In this cohort, 9% of men and 13% of women had concentrations <30 nmol/L, and 9% of men and 8% of women had concentrations >90 nmol/L. Plasma 25(OH)D concentrations were inversely related to BMI, current smoking status, and physical inactivity and positively related to lung function and blood vitamin C concentrations.

**TABLE 1:** Descriptive characteristics of 6486 men and 8155 women in the EPIC-Norfolk 1997–2000 by serum 25(OH)D [total of and 25(OH)D_2_ and 25(OH)D_3_] category*^1^*

	Plasma 25(OH)D category (nmol/L)					
	<30	30 to <50	50 to <70	70 to <90	≥90	*P* (ANOVA)
Men						
* n*	603	1952	2138	1240	553	—
Total 25(OH)D (nmol/L)	23.3 ± 4.9*^2^*	40.9 ± 5.7	59.5 ± 5.6	78.5 ± 5.6	105.5 ± 15.0	<0.0001
25(OH)D_3_ (nmol/L)	23.1 ± 4.9	40.7 ± 5.9	59.2 ± 5.9	78.3 ± 5.8	104.7 ± 15.9	<0.0001
25(OH)D_2_ (nmol/L)	0.2 ± 0.9	0.3 ± 1.4	0.3 ± 1.5	0.3 ± 1.3	0.8 ± 5.3	<0.0001
3-epi-25(OH)D_3_ (nmol/L)	0.6 ± 1.0	1.0 ± 1.3	1.5 ± 1.4	2.1 ± 1.7	3.1 ± 2.4	<0.0001
Age (y)	63.3 ± 9.4	63.2 ± 9.0	63.0 ± 9.1	62.4 ± 8.8	62.7 ± 8.7	<0.001
BMI (kg/m^2^)	27.4 ± 3.8	27.2 ± 3.5	26.9 ± 3.2	26.6 ± 3.1	26.3 ± 2.9	<0.001
Systolic blood pressure (mm Hg)	138.6 ± 17.5	138.0 ± 18.0	137.3 ± 16.8	135.9 ± 16.8	135.7 ± 16.2	0.001
Cholesterol (mmol/L)	5.8 ± 1.1	5.8 ± 1.1	5.9 ± 1.1	5.9 ± 1.1	6.0 ± 1.1	0.03
Respiratory function FEV_1_ (cL/s)	270 ± 82	282 ± 76	290 ± 71	291 ± 72	298 ± 73	<0.0001
Vitamin C (nmol/L)	50.6 ± 21.5	54.8 ± 22.0	57.5 ± 20.8	60.1 ± 20.1	58.5 ± 19.5	<0.001
Alcohol (units/d)	2.5 ± 1.8	2.5 ± 1.7	2.6 ± 1.7	2.6 ± 1.7	2.9 ± 1.7	<0.001
Dietary calcium intake (mg/d)	1030 ± 314	1051 ± 296	1056 ± 293	1066 ± 309	1040 ± 294	0.15
Physically inactive [% (*n*)]	34.9 (210)	28.0 (547)	24.1 (515)	22.7 (281)	18.6 (103)	<0.001
Manual social class [% (*n*)]	34.1 (201)	37.8 (728)	38.2 (804)	39.9 (489)	39.3 (213)	0.19
No educational qualifications [% (*n*)]	24.9 (150)	27.4 (535)	28.1 (598)	28.2 (350)	26.2 (145)	0.55
Current smokers [% (*n*)]	15.4 (92)	8.4 (162)	6.5 (138)	6.5 (80)	8.6 (47)	<0.001
History of cardiovascular disease [% (*n*)]	7.3 (44)	6.5 (126)	5.0 (107)	5.9 (73)	4.3 (20)	0.07
History of diabetes [% (*n*)]	5.7 (32)	5.4 (99)	4.1 (80)	3.6 (42)	4.1 (21)	0.06
History of bronchitis or asthma [% (*n*)]	15.4 (93)	14.2 (277)	14.1 (301)	12.5 (155)	12.7 (70)	0.39
History of any fracture [% (*n*)]	6.0 (36)	5.1 (100)	6.0 (128)	5.8 (72)	6.0 (33)	0.79
Women						
* n*	1037	2594	2564	1340	620	—
Total 25(OH)D (nmol/L)	23.2 ± 5.1	40.6 ± 5.7	59.4 ± 5.7	78.7 ± 5.6	104.3 ± 14.5	<0.0001
25(OH)D_3_ (nmol/L)	23.0 ± 5.1	40.2 ± 5.9	59.0 ± 5.9	78.2 ± 6.1	103.8 ± 14.6	<0.0001
25(OH)D_2_ (nmol/L)	0.2 ± 0.9	0.4 ± 1.8	0.4 ± 1.8	0.5 ± 2.1	0.5 ± 2.3	<0.0001
3-epi-25(OH)D_3_ (nmol/L)	0.6 ± 1.0	0.9 ± 1.5	1.3 ± 1.5	1.7 ± 1.4	2.5 ± 1.8	<0.0001
Age (y)	62.9 ± 9.5	62.3 ± 9.3	61.3 ± 8.8	60.4 ± 8.7	59.0 ± 8.2	<0.001
BMI (kg/m^2^)	27.3 ± 5.0	26.9 ± 4.6	26.4 ± 4.1	25.8 ± 3.8	25.3 ± 3.7	<0.001
Systolic blood pressure (mm Hg)	135.6 ± 18.9	134.7 ± 18.6	133.1 ± 18.3	131.2 ± 18.0	129.1 ± 17.8	<0.001
Cholesterol (mmol/L)	6.2 ± 1.2	6.2 ± 1.2	6.2 ± 1.2	6.2 ± 1.2	6.2 ± 1.2	0.84
Respiratory function FEV1 (cL/s)	203 ± 57	209 ± 52	216 ± 51	221 ± 52	226 ± 49	<0.0001
Vitamin C (nmol/L)	62.2 ± 25.4	67.2 ± 25.1	70.2 ± 24.7	70.5 ± 20.5	71.8 ± 20.6	<0.001
Alcohol (units/d)	1.5 ± 1.2	1.6 ± 1.2	1.7 ± 1.3	1.8 ± 1.3	1.8 ± 1.3	<0.001
Dietary calcium intake (mg/d)	992 ± 279	1003 ± 293	1011 ± 288	1000 ± 283	983 ± 302	0.21
Physically inactive [% (*n*)]	34.5 (357)	26.7 (691)	22.2 (570)	19.4 (260)	17.3 (107)	<0.001
Manual social class [% (*n*)]	37.6 (379)	35.3 (899)	36.7 (922)	34.7 (458)	36.9 (225)	0.51
No qualifications [% (*n*)]	43.1 (447)	42.6 (1105)	43.9 (1126)	42.9 (575)	41.6 (258)	0.82
Current smokers [% (*n*)]	11.8 (121)	8.0 (205)	7.1 (181)	6.5 (86)	10.1 (62)	<0.001
History of cardiovascular disease [% (*n*)]	2.5 (26)	2.4 (63)	1.6 (42)	0.8 (11)	1.1 (7)	0.001
History of diabetes [% (*n*)]	3.6 (31)	3.0 (64)	2.4 (56)	2.5 (31)	2.1 (13)	0.26
History of bronchitis or asthma [% (*n*)]	16.9 (175)	15.4 (400)	15.8 (405)	15.3 (205)	15.6 (97)	0.84
History of any fracture [% (*n*)]	7.3 (76)	8.1 (211)	7.5 (192)	6.3 (85)	6.6 (41)	0.31

1 Total 25(OH)D comprises the sum of 25(OH)D_2_ and 25(OH)D_3_. Data on the C3 epimer shown for information but not included in the total. Conversion factors for nanomoles per liter to nanograms per milliliter were as follows: for 25(OH)D_3_, divide nanomoles per liter by 2.496 to obtain nanograms per milliliter; for 25(OH)D_2_, divide nanomoles per liter by 2.42 to obtain nanograms per milliliter. As a general approximation, multiply nanomoles per liter by 0.4 to obtain nanograms per milliliter (30 nmol/L = 12 ng/mL, 50 nmol/L = 20 ng/mL, 70 nmol/L = 28 ng/mL, 90 nmol/L = 36 ng/mL, and 120 nmol = 48 ng/mL). EPIC-Norfolk, European Prospective Investigation into Cancer and Nutrition–Norfolk; FEV_1_, forced expiratory volume in 1 s; 25(OH)D, 25-hydroxyvitamin D.

2 Mean ± SD (all such values).

Mortality rates from 1997 to 2000 to 2012 and HRs by plasma 25(OH)D category by cause adjusted for age, sex, and month of blood draw and additionally adjusted for BMI, cigarette smoking, physical activity, alcohol intake, plasma vitamin C, social class, education, diabetes, history of cardiovascular disease, and history of cancer are shown in **Table 2**. HRs for incident total cancer, incident cardiovascular diseases, respiratory diseases, total fractures, and hip fractures adjusted first for age, sex, and month and further multivariable adjusted as described previously are also shown in Table 2. There was a strong inverse association between increasing 25(OH)D category and total mortality with individuals with concentrations >90 nmol/L having 34% lower risk of mortality compared than for subjects with concentrations <30 nmol/L. There appeared to be a graded relation across the distribution of vitamin D concentrations. Trends were apparent for deaths from cardiovascular causes and respiratory disease causes with the greatest magnitude of difference for respiratory causes but not significant for deaths from cancer or other noncardiovascular nonrespiratory underlying causes. When analyzed as a continuous variable, for every 20-nmol/L increase in 25(OH)D, there was 11% lower cardiovascular disease mortality risk and 30% lower respiratory disease mortality risk. These associations only slightly attenuated after multivariable adjustment.

**TABLE 2:** Rates and HRs by serum 25(OH)D category for mortality by cause and hospitalization for cardiovascular disease, respiratory disease, and fractures in 14,641 men and women in the EPIC-Norfolk 1997–2012*^1^*

	Serum 25(OH)D category (nmol/L)						
	<30 (*n* = 1640)	30 to <50 (*n* = 4546)	50 to <70 (*n* = 4702)	70 to <90 (*n* = 2580)	≥90 (*n* = 1173)	HR (95% CI) per 20-nmol/L increase in serum 25(OH)D	*P*-linear trend
Deaths: all causes							
% (*n*)	24.2 (397)	21.2 (966)	17.7 (834)	16.1 (416)	13.9 (163)	—	<0.0001
HR (95% CI)*^2^*	1	0.84 (0.74, 0.94)	0.72 (0.63, 0.81)	0.71 (0.62, 0.82)	0.66 (0.55, 0.79)	0.89 (0.86, 0.93)	<0.0001
HR (95% CI)*^3^*	1	0.90 (0.79, 1.03)	0.78 (0.68, 0.90)	0.80 (0.68, 0.94)	0.73 (0.59, 0.90)	0.92 (0.88, 0.96)	<0.0001
Deaths from cardiovascular causes							
% (*n*)	7.7 (126)	6.8 (308)	5.4 (254)	4.7 (121)	3.8 (45)	—	<0.0001
HR (95% CI)*^2^*	1	0.86 (0.70, 1.06)	0.72 (0.58, 0.90)	0.70 (0.54, 0.91)	0.62 (0.44, 0.88)	0.88 (0.83, 0.94)	<0.0001
HR (95% CI)*^3^*	1	0.95 (0.74, 1.21)	0.84 (0.65, 1.09)	0.82 (0.61, 1.11)	0.73 (0.49, 1.09)	0.92 (0.85, 0.99)	0.03
Deaths from cancer causes							
% (*n*)	7.7 (126)	8.3 (376)	7.0 (331)	6.9 (179)	6.3 (74)	—	0.014
HR (95% CI)*^2^*	1	0.97 (0.79, 1.19)	0.85 (0.79, 1.05)	0.86 (0.68, 1.09)	0.85 (0.62, 1.09)	0.93 (0.88, 0.99)	0.017
HR (95% CI)*^3^*	1	1.11 (0.89, 1.40)	0.94 (0.74, 1.19)	0.96 (0.73, 1.25)	0.90 (0.65, 1.26)	0.94 (0.89, 1.00)	0.07
Deaths from respiratory causes							
% (*n*)	3.4 (56)	1.8 (81)	1.3 (60)	1.2 (30)	0.7 (8)	—	<0.0001
HR (95% CI)*^2^*	1	0.47 (0.34, 0.67)	0.34 (0.24, 0.50)	0.35 (0.22, 0.56)	0.22 (0.10, 0.46)	0.70 (0.62, 0.81)	<0.0001
HR (95% CI)*^3^*	1	0.48 (0.33, 0.70)	0.36 (0.24, 0.55)	0.42 (0.26, 0.69)	0.24 (0.11, 0.54)	0.73 (0.63, 0.85)	<0.0001
Deaths from other causes (noncardiovascular, noncancer, and nonrespiratory)							
% (*n*)	5.4 (89)	4.4 (201)	4.0 (189)	3.3 (86)	3.1 (36)		<0.0001
HR (95% CI)*^2^*	1	0.79 (0.61, 1.01)	0.74 (0.57, 0.96)	0.69 (0.51, 0.94)	0.69 (0.46, 1.02)	0.91 (0.84, 0.99)	0.02
HR (95% CI)*^3^*	1	0.83 (0.62, 1.11)	0.79 (0.58, 1.07)	0.84 (0.59, 1.18)	0.82 (0.53, 1.28)	0.96 (0.88, 1.05)	0.38
All cancer incidence							
% (*n*)	21.0 (345)	22.0 (999)	21.2 (996)	21.2 (546)	20.0 (235)	—	0.25
HR (95% CI)*^2^*	1	0.97 (0.86, 1.10)	0.95 (0.84, 1.08)	1.05 (0.91, 1.20)	1.00 (0.85, 1.19)	1.01 (0.97, 1.04)	0.77
HR (95% CI)*^3^*	1	1.00 (0.88, 1.15)	1.02 (0.89, 1.17)	1.12 (0.96, 1.31)	1.08 (0.89, 1.30)	1.02 (0.99, 1.06)	0.21
Cardiovascular disease events							
% (*n*)	36.2 (593)	3331 (1561)	29.2 (1372)	27.3 (704)	24.2 (284)	—	<0.0001
HR (95% CI)*^2^*	1	0.91 (0.82, 0.99)	0.80 (0.73, 0.89)	0.78 (0.70, 0.87)	0.75 (0.70, 0.88)	0.92 (0.90, 0.95)	<0.0001
HR (95% CI)*^3^*	1	0.96 (0.85, 1.07)	0.87 (0.78, 0.98)	0.90 (0.79, 1.02)	0.89 (0.75, 1.05)	0.96 (0.93, 0.99)	0.014
Respiratory disease events							
% (*n*)	21.0 (345)	15.8 (718)	13.2 (621)	12.4 (321)	10.8 (127)	—	<0.0001
HR (95% CI)*^2^*	1	0.72 (0.63, 0.82)	0.59 (0.51, 0.68)	0.57 (0.48, 0.67)	0.55 (0.44, 0.68)	0.85 (0.82, 0.90)	<0.0001
HR (95% CI)*^3^*	1	0.73 (0.62, 0.84)	0.61 (0.52, 0.72)	0.65 (0.54, 0.78)	0.59 (0.46, 0.75)	0.89 (0.84, 0.93)	<0.0001
All fracture events							
% (*n*)	5.4 (89)	4.2 (189)	3.5 (165)	3.3 (86)	2.9 (34)	—	<0.0001
HR (95% CI)*^2^*	1	0.77 (0.59, 0.99)	0.68 (0.52, 0.90)	0.65 (0.47, 0.89)	0.62 (0.40, 0.95)	0.88 (0.81, 0.96)	0.002
HR (95% CI)*^3^*	1	0.78 (0.58, 1.05)	0.70 (0.51, 0.95)	0.65 (0.45, 0.93)	0.64 (0.40, 1.03)	0.89 (0.81, 0.97)	0.012
Hip-fracture events							
% (*n*)	2.2 (36)	1.6 (72)	1.2 (58)	0.7 (19)	1.1 (13)	—	<0.0001
HR (95% CI)*^2^*	1	0.76 (0.51, 1.14)	0.65 (0.42, 1.01)	0.41 (0.22, 0.74)	0.65 (0.34, 1.33)	0.81 (0.79, 0.94)	0.004
HR (95% CI)*^3^*	1	0.67 (0.42, 1.07)	0.60 (0.37, 0.98)	0.38 (0.20, 0.74)	0.55 (0.25, 1.23)	0.78 (0.66, 0.93)	0.004

1 Conversion factors for nanomoles per liter to nanograms per milliliter were as follows: for 25(OH)D_3_, divide nanomoles per liter by 2.496 to obtain nanograms per milliliter; for 25(OH)D_2_, divide nanomoles per liter by 2.42 to obtain nanograms per milliliter. As a general approximation, multiply nanomoles per liter by 0.4 to obtain nanograms per milliliter (30 nmol/L = 12 ng/mL, 50 nmol/L = 20 ng/mL, 70 nmol/L = 28 ng/mL, 90 nmol/L = 36 ng/mL, and 120 nmol = 48 ng/mL). EPIC-Norfolk, European Prospective Investigation into Cancer and Nutrition–Norfolk; 25(OH)D, 25-hydroxyvitamin D.

2 Age, sex, and month adjusted.

3 Age, sex, month, BMI, physical activity, smoking, alcohol, vitamin C, diabetes, history of cardiovascular disease, history of cancer, social class, and education adjusted.

Consistent with the mortality findings, baseline 25(OH)D concentrations were not significantly associated with cancer incidence but were inversely associated with incident total cardiovascular disease and respiratory disease with the greatest magnitude of association observed for respiratory diseases. Concentrations of 25(OH)D were also significantly inversely associated with incident fractures and hip fractures although less apparently linearly with the lower RR flattening at concentrations >50 nmol/L.

HRs for total mortality per increase of 20 nmol/L serum 25(OH)D, which were consistent in the various subgroups examined, including stratification by dietary calcium intake and also apparent after the exclusion individuals with prevalent diseases or individuals who died ≤2 or ≤5 y follow-up are shown in **Table 3**.

**TABLE 3:** Cox multivariable-adjusted HRs for all-cause mortality in men and women in the EPIC-Norfolk 1997–2012 per 20-nmol/L increase in serum 25(OH)D concentrations in subgroups*^1^*

	*n*/Total *n*	HR (95% CI) per 20-nmol/L increase in serum 25(OH)D (age, sex, and month adjusted)	*P*	HR (95% CI) per 20-nmol/L increase in serum 25(OH)D (age, sex, month, and multivariable adjusted)*^2^*	*P*
All	2776/14,641	0.89 (0.86, 0.93)	<0.0001	0.92 (0.88, 0.96)	<0.0001
By sex					
Men	1589/6486	0.89 (0.85, 0.94)	<0.0001	0.92 (0.87, 0.97)	0.001
Women	1187/8187	0.89 (0.84, 0.94)	<0.0001	0.92 (0.86, 0.98)	0.011
By age					
<65 y	618/8578	0.88 (0.81, 0.94)	<0.0001	0.92 (0.85, 0.99)	0.038
≥65 y	2158/6060	0.90 (0.86, 0.94)	<0.0001	0.92 (0.88, 0.97)	0.001
Excluding early deaths					
Excluding deaths ≤2 y	2597/14,459	0.90 (0.87, 0.93)	<0.0001	0.92 (0.89, 0.97)	<0.0001
Excluding deaths ≤5 y	2135/13,997	0.91 (0.88, 0.95)	<0.001	0.94 (0.89, 0.98)	<0.001
By smoking status					
Current smokers	339/1332	0.89 (0.81, 0.98)	0.018	0.90 (0.80, 1.01)	0.066
Current nonsmokers	2442/13,347	0.91 (0.87, 0.94)	<0.0001	0.93 (0.89, 0.97)	0.001
By BMI					
<27 kg/m^2^	1496/8557	0.89 (0.85, 0.94)	<0.0001	0.92 (0.87, 0.97)	0.002
≥27 kg/m^2^	1267/6057	0.91 (0.86, 0.96)	<0.001	0.93 (0.88, 0.99)	0.030
By physical activity					
Physically inactive	1042/3641	0.88 (0.83, 0.93)	<0.0001	0.90 (0.84, 0.97)	0.003
Not physically inactive	1734/10,093	0.92 (0.88, 0.967	<0.001	0.94 (0.89, 0.98)	0.009
Excluding persons with a history of heart disease, stroke, cancer, diabetes, or fracture	1872/11,676	0.90 (0.86, 0.93)	<0.001	0.92 (0.88, 0.96)	<0.001
Excluding persons with history of asthma or bronchitis	2284/12,463	0.90 (0.87, 0.93)	<0.0001	0.92 (0.89, 0.96)	<0.001
By social class					
Nonmanual social class	1695/9067	0.88 (0.84, 0.93)	<0.0001	0.91 (0.86, 0.95)	<0.0001
Manual social class	1001/5318	0.92 (0.87, 0.98)	0.008	0.95 (0.88, 1.01)	0.12
By vitamin supplement use					
No vitamin supplement use	1177/6604	0.91 (0.86, 0.96)	<0.0001	0.92 (0.86, 0.98)	0.007
Any vitamin supplement use	1421/7317	0.90 (0.85, 0.95)	<0.0001	0.93 (0.87, 0.98)	0.010
By dietary calcium intake					
<1000 mg/d	1302/6754	0.91 (0.86, 0.96)	0.02	0.93 (0.87, 0.99)	0.02
≥1000 mg/d	1284/5628	0.88 (0.84, 0.93)	<0.0001	0.91 (0.86, 0.97)	0.004

1 Conversion factors for nanomoles per liter to nanograms per milliliter were as follows: for 25(OH)D_3_, divide nanomoles per liter by 2.496 to obtain nanograms per milliliter; for 25(OH)D_2_, divide 25(OH)D_2_ by 2.42 to obtain nanograms per milliliter. As a general approximation, multiply nanomoles per liter by 0.4 to obtain nanograms per milliliter (30 nmol/L = 12 ng/mL, 50 nmol/L = 20 ng/mL, 70 nmol/L = 28 ng/mL, 90 nmol/L = 36 ng/mL, and 120 nmol = 48 ng/mL). EPIC-Norfolk, European Prospective Investigation into Cancer and Nutrition–Norfolk; 25(OH)D, 25-hydroxyvitamin D.

2 Further adjusted for BMI, cigarette smoking, alcohol intake, plasma vitamin C, physical activity, diabetes, history of cardiovascular disease, history of cancer, social class, and educational level (except where the variable was used for stratification). Numbers do not always add up to total because of missing data.

**FIGURE 1.**
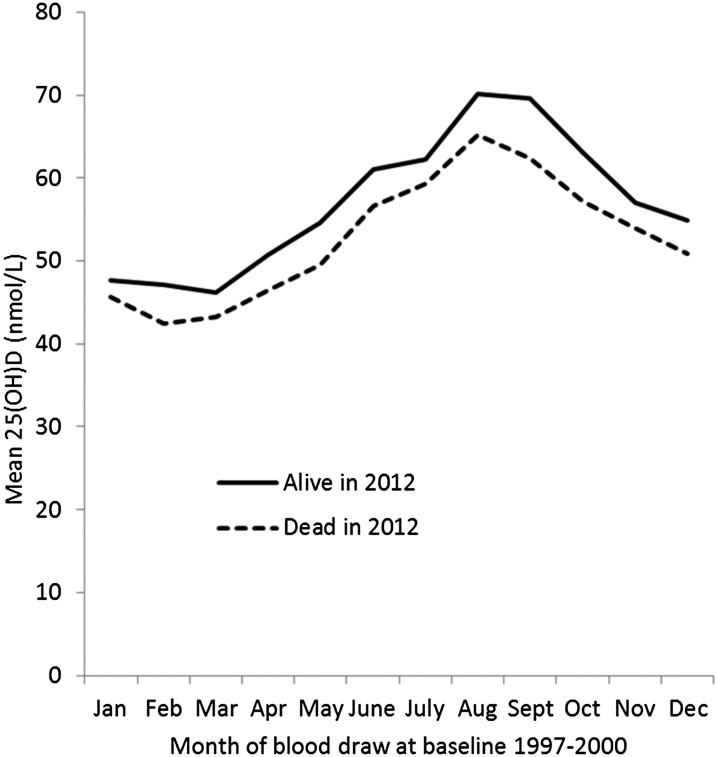
Mean serum 25(OH)D concentrations by month at the 1997–2000 baseline examination in subjects alive and subjects dead at follow-up in 2012. The mean difference in baseline 25(OH)D concentrations in subjects alive compared with subjects dead in 2012, adjusted by age, sex, and month was +3.8 nmol/L (*P* < 0.0001). Estimated coefficients in nanomoles per liter to add to 25(OH)D concentration in nanomoles per liter to adjust for the month of blood draw to characterize the individual annual mean were as follows: Jan, +9; Feb, +11; Mar, +11; Apr, +6; May, +3; June, −4; July, −5; Aug, −12, −Sep, −11; Oct, −5; Nov, (mean); Dec, +2. 25(OH)D, 25-hydroxyvitamin D.

Mean baseline vitamin D concentrations by the month of blood draw in 1993–1997 in individuals who were dead compared with those who were alive at the end of follow-up in 2012 are shown in **Figure 1**. Despite a substantial seasonal variation in mean vitamin D concentrations, there was a consistent difference throughout the months between individuals who died and those who survived over the 13 subsequent years of follow-up. Coefficients for the month of blood draw to obtain the estimated mean annual vitamin D for categorizing individuals are presented in the figure legend.

We previously reported an association between 25(OH)D concentrations and incident diabetes on the basis of a nested case-control design by using blood samples from the 1993–1997 baseline survey taken 4 y before the current study (8). The 25(OH)D assays for this earlier analysis were undertaken in a different laboratory by using different methods. Nevertheless, in 877 individuals with available vitamin D measures in both 1993–1997 and 1997–2000, there was a correlation of 0.36 (*P* < 0.001) between the 2 independent samples. After adjustment for the month of blood draw at both visits by using the coefficients shown in Figure 1, the correlation was 0.45 (*P* < 0.001).

**FIGURE 2.**
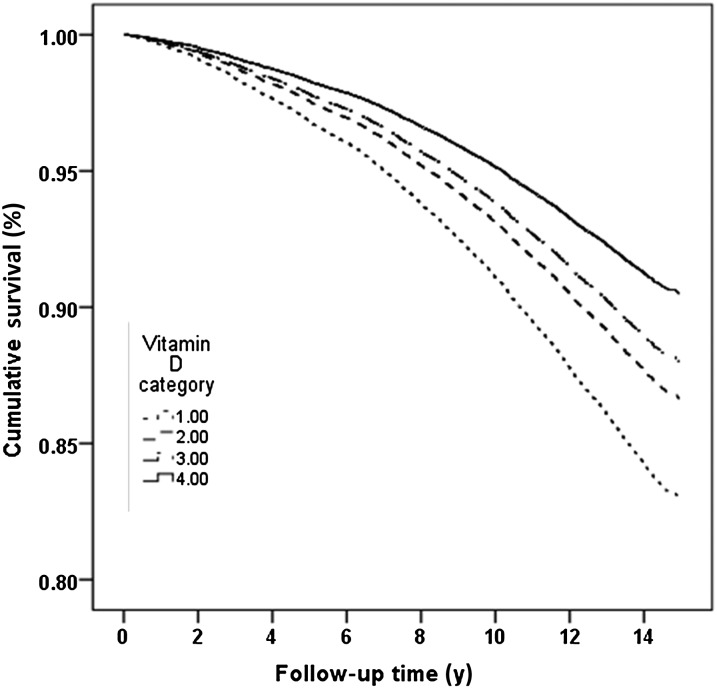
Survival between 1997 and 2012 by serum 25-hydroxyvitamin D category in men and women with adjustment for age, sex, and month.

Only 2 individuals had 25(OH)D concentrations >180 nmol/L (>72 ng/mL), which is the recommended upper limit. There were no deaths that occurred in this group. We recategorized the cohort according to new cutoffs with an upper group of ≥120 nmol/L (48 ng/mL) to explore the association with mortality at the higher concentrations of 25(OH)D although numbers were small in this high category (*n* = 152; 1% of the total cohort). Survival curves over follow-up between 1997 and 2012 are shown in **Figure 2**. Age-, sex-, and month-adjusted HRs (95% CIs) for cutoffs <30, 30 to <90, 90 to <120, and ≥120 nmol/L were 1, 0.77 (0.69, 0.86), 0.68 (0.56, 0.83), and 0.53 (0.33, 0.87), respectively. Analyses conducted by using only 25(OH)D_3_ rather than total 25(OH)D showed essentially similar results.

## DISCUSSION

In these middle-aged and older men and women, plasma 25(OH)D concentrations measured between 1997 and 2000 were inversely associated with total mortality over an average of 13 y of follow-up. There appeared to be a dose-response relation across the whole distribution of 25(OH)D concentrations with the lowest mortality observed in the category with blood concentrations >90 nmol/L. Approximately 10% of the population had concentrations <30 mmol/L, 9% of the population had concentrations >90 nmol/L, and 1% of the population had concentrations >120 nmol/L. This inverse association was consistent after adjustment for age, sex, month of blood draw, BMI, physical activity, smoking, alcohol intake, plasma vitamin C concentrations, occupational social class, education, diabetes history, history of cardiovascular disease, and history of cancer. This association was also consistent in subgroups after stratification for age, sex, smoking status, BMI, physical activity, any vitamin supplement use, social class, and dietary calcium intake and after exclusions of individuals with prevalent chronic diseases and deaths within the first 5 y. Across the observed range of 25(OH)D concentrations in this cohort, each increase of 20 nmol/L in blood concentrations was associated with ∼8% lower observed mortality risk over the 13 y follow-up. The association was strongest for mortality from respiratory diseases. Plasma 25(OH)D was also inversely associated with incident cardiovascular disease and respiratory disease and also for total fractures and hip fractures. Again, strongest associations were observed for respiratory diseases. Plasma 25(OH)D was not associated with total cancer incidence.

Substantial debate continues as to what optimal vitamin D status might be above low blood concentrations associated with deficiency and bone diseases. An Institute of Medicine report set serum 25(OH)D cutoffs of increased risk of deficiency at <30 nmol/L, adequacy at >50 nmol/L, and increased risk of excess at >125 nmol/L on the basis of reviews that suggested a reverse J curve for overall mortality with highest risk from low vitamin D (<30 nmol/L), lower risk between vitamin D concentrations of 30–60 nmol/L, with risk increasing again at higher concentrations [>75 nmol/L in whites and >60 nmol in African Americans and adverse outcomes with serum 25(OH)D concentration >125 nmol/L (1)]. Although the European Society for Clinical and Economic Aspects of Osteoporosis and Arthritis stated that, above a serum threshold of 50 nmol/L for 25(OH)D concentrations, there is no clear evidence for additional benefits of supplementation, they indicated that, in fragile elderly subjects at elevated risk of falls and fractures, a minimal serum 25(OH)D concentration of 75 nmol/L is recommended (28).

Randomized clinical trials of vitamin D supplementation have not provided conclusive evidence. A review of 56 randomized trials with 95286 participants that examined vitamin D at any dose, duration, and route of administration observed that vitamin D in the form of vitamin D_3_ seems to decrease mortality in predominantly elderly women who are mainly in institutional care but otherwise concluded that the available evidence on vitamin D supplementation and mortality is inconclusive (2). A similar debate applies to vitamin D–supplementation trials for prevention of fractures, cardiovascular disease, and other health outcomes (1, 2, 5, 16, 29–33).

Some of the uncertainty has been attributed variously to the heterogeneity in the baseline vitamin D status of the populations studied, uncertain compliance, different modes of administration of vitamin D because oral compared with intramuscular administration or small daily doses compared with infrequent bolus doses result in very different blood concentrations (34), different forms of vitamin D administered because different isomers such as cholecalciferol (vitamin D_3_) and ergocalciferol (vitamin D_2_) are postulated to have different biological potency and effects (35), possible interactions with other nutrients such as dietary or supplemental calcium (7), and low power for mortality. Many trials have focused on high-risk groups such as trials of the prevention of fractures in the very old or individuals with osteoporosis, and the largest trial to date, the Women's Health Initiative, was conducted only in postmenopausal women (33).

Observational studies, particularly cohort studies, may be better placed to quantify the association between vitamin D and health outcomes, but results have also been inconsistent (14, 36). Vitamin D is manufactured in the skin in the presence of sunlight, and thus, blood concentrations rather than the assessment of dietary intake provide the most accurate assessment of individual vitamin D status. Hitherto, the assay of 25(OH)D concentrations in large-population cohorts has been challenging for reasons of cost and assay variations. Many assays have not distinguished between the various isomers of vitamin D such as 25(OH)D_3_, 25(OH)D_2_, and the C3 epimer 3-epi-25(OH)D_3_ (37) and variations in assay methods make the interpretation of and comparison between studies difficult (38). Nevertheless a systematic review of 12 prospective studies in 32142 individuals of mainly elderly study participants with measured 25(OH)D in whom 6921 died during follow-up showed an inverse association between 25(OH)D and all-cause mortality (14). The meta analysis estimated a pooled HR of 0.92 (95% CI: 0.89, 0.95) for a 25-nmol/L increase in 25(OH)D concentrations, which is an estimate that is remarkably similar to that observed in the current single cohort of 14,679 individuals with 2780 deaths. These results are also compatible with results from a 5-y vitamin D_3_–supplement randomized trial with means of 74.3 compared with 53.4 nmol/L in vitamin D–supplement and placebo groups, respectively, resulting in a mean difference of ∼21 nmol/L and a nonsignificant RR of 0.88 (95% CI: 0.74, 1.06; *P* = 0.18) of mortality in supplement compared with placebo groups (39).

We also showed strong inverse associations of serum 25(OH)D with incident and fatal cardiovascular disease, respiratory disease, and fractures. Although these associations have been variably documented previously (6, 11, 13, 40–43), the dose-response relation between plasma 25(OH)D concentrations and health outcomes and the nature of the relationship, whether threshold or U-shaped, are still debated.

This study, which included men and women, enabled the examination of the dose-response relation with total mortality at concentrations >90 and >120 nmol/L although with only a small proportion of the population with concentrations >120 nmol/L (*n* = 152; 1%) and >150 nmol (*n* = 21; 0.1%); we did not have power to assess potential adverse outcomes above concentrations of 150 nmol/L. Nevertheless, mean 25(OH)D distributions and seasonal variations were broadly comparable to those noted for other Northern hemisphere countries, most notably in the US NHANES III.

The significant relations were somewhat surprising because only one measure of 25(OH)D was used to characterize individual status, and large seasonal variations in 25(OH)D concentrations have been well established. Nevertheless, comparisons of mean vitamin D concentrations by the baseline month of blood draw in subjects who survived and those who were alive after 13 y of follow-up showed consistent significant differences in mean concentrations throughout the year. A random measurement error from intraindividual variation was likely to have attenuated any associations with endpoints.

Because of the large seasonal variation, characterizing an individual's usual vitamin D status is challenging, and various algorithms of varying complexity have been proposed (44); our data suggested that a simple adjustment on the basis of the month of blood draw outlined in Figure 1 is adequate for these purposes in this UK population.

Many assays do not distinguish between various 25(OH)D isomers. With the use of a high-sensitivity assay, the predominant form of vitamin D was vitamin D_3_ (97%), and the other isomers 25(OH)D_2_ and 3-epi-25(OH)D_3_ only contributed a small proportion. Main results are shown for total 25(OH)D but were very similar for 25(OH)D_3_ alone. We were unable to assess whether 25(OH)D_2_ derived from ergocalciferol would have similar associations.

Incident diseases were ascertained from mortality and hospital-admission data, which in the United Kingdom with the National Health Service are virtually complete. Validation studies on the basis of medical records have shown good validation for cardiovascular diseases. However, milder nonfatal cases that did not require a hospital admission would have been missed. Although this absence may have limited the generalizability of results to more-severe diseases, it is likely that a measurement error in disease ascertainment would have attenuated associations.

In conclusion, this 13-y prospective study in a free-living, middle-aged and older British population provides additional support for the hypothesis that vitamin D status is associated with a range of important health outcomes including respiratory disease, cardiovascular disease, fractures, and total mortality. Highest mortality rates were observed in individuals with 25(OH)D concentrations <30 nmol/L. Negligible numbers of subjects had concentrations >150 nmol/L, and only 1% of the population had concentrations >120 nmol/L. Within this observed population range, there was no evidence for increased mortality for 25(OH)D >90 or >120 nmol/L, suggesting that a moderate increase in population mean concentrations may have a potential health benefit for preventing deficiency without increasing risk.
